# Management of Salter–Harris Type 1 Fracture Complicated with Osteomyelitis in a Sickle Cell Disease Patient: A Case Report and Review of Literature

**DOI:** 10.3390/medicines9100050

**Published:** 2022-09-22

**Authors:** Nnennaya U. Opara, Emmanuella C. Osuala, Ugochinyere I. Nwagbara

**Affiliations:** 1 Department of Emergency Medicine, Charleston Area Medical Center Institute for Academic Medicine, Charleston, WV 25304, USA; 2 Department of Health Administration, University of Phoenix, Phoenix, AZ 85040, USA; 3 Department of Pharmaceutical Sciences, College of Health Sciences, University of KwaZulu-Natal Westville Campus, Durban 4000, South Africa; 4 Department of Public Health Medicine, College of Health Sciences, University of KwaZulu-Natal, Howard Campus, Durban 4041, South Africa

**Keywords:** Salter–Harris type, sickle-cell disease, osteomyelitis, fracture

## Abstract

Salter–Harris fractures may occur due to a single injury or repetitive stress fractures on the extremities. Type I to III fractures are managed medically, while types IV and V, which are rare, are treated surgically. In the pediatric population, Salter–Harris I fractures of the distal tibia are commonly seen, and management of such fractures are well established in the literature. Despite the availability of a wide range of treatment for such fractures, osteonecrosis or avascular necrosis of the proximal femur can subsequently develop. Avascular necrosis is cell death secondary to metabolic disturbances, trauma, adverse effects of certain medications, or sickle cell disease. Avascular necrosis commonly affects the talus, humerus, or tibia in addition to the femoral head. Radiographic images are essential for prompt diagnosis and to minimize negative health outcomes in these patients. However, Salter–Harris I fracture in sickle cell patients can be very challenging due to these patients’ vulnerability to bone infections and sickle cell crisis. In this case report, our patient with a history of sickle cell disease and with a diagnosis of Salter–Harris I fracture was treated with surgical intervention as type V, which is discussed in this article, and responded well to treatment. Thus, this case suggests a new approach to managing Salter–Harris I fractures complicated with osteomyelitis in sickle cell patients.

## 1. Introduction

Salter–Harris fractures are often sports-related injuries among older children. However, they can also be seen as a result of child abuse, injury from frostbite, radiation, and neurological disorders, and, in some cases, can be medication-induced, all affecting the growth plate. Most Salter–Harris I fractures are managed with non-invasive procedures (cast immobilization). This is different when managing similar fractures in sickle cell disease patients due to their high susceptibility to infections, particularly osteomyelitis.

Sickle cell disease, an inherited autosomal recessive disorder, is a form of a hemolytic anemia due to abnormally shaped (sickled) red blood cells (RBCs), which are removed from the blood circulation and are destroyed in the spleen at increased rates, resulting in anemia. According to the Global Burden of Disease Study [1], approximately 3.2 million people are affected with sickle cell disease, while 43 million people are carriers, and 176 thousand people die annually from complications from sickle cell disease. The most common form of sickle cell disease is sickle cell anemia, which accounts for 70% of the cases of sickle cell disease in Africans [2]. Sickle cell anemia is caused by homozygosity of the beta-S allele located on chromosome 11p15.5, which differs from the wild type beta-allele by a single nucleotide polymorphism Rs334(T:T) where GTG (Val) is substituted for GAG (Glu) in the sixth codon of the beta-globin gene [2,3,4,5], thus forming a mutated hemoglobin tetramer HbS in the red blood cells of patients with sickle cell anemia [6,7]. The sickled red blood cells occur because of a single amino acid substitution in the beta-globin chain which forms hemoglobin S and, when deoxygenated, results in a sickle-shaped red blood cell. The major cause of morbidity in these patients is acute vaso-occlusive crisis due to microvascular occlusion by the sickled red blood cells resulting in tissue ischemia and bone infarction [8]. The involvement of the bone is common, which is manifested as a painful vaso-occlusive crisis and progressive disabilities due avascular necrosis. Other bone involvement in sickle cell disease patients include osteomyelitis [9], orbital bone infarction [10], dental issues [11], dactylitis, osteoporosis, and osteopenia. Sickle cell disease is associated with several other health conditions, including hyposplenism, and impaired complement activity. The management of sickle cell disease patients with Salter–Harris I fracture can be challenging, particularly in the pediatric population. In this case report, we report the case of a 17-year-old sickle cell disease patient with Salter–Harris fracture type I secondary to a fall who was effectively treated for his fractures with a unique approach.

## 2. Case Presentation

A previously healthy 17-year-old African male with sickle cell disease complaining of persistent pain secondary to the fracture of the distal end of the left femur for 6 months was referred to a specialist hospital by his family physician. The patient (a pianist and high school graduate) was in good health condition prior to having a nasty fall and was unable to bear weight on his left leg. He was initially treated by his family physician, but when he was unable to recover fully after 6 months of pain medications and a plaster cast, he was referred to a specialist hospital for proper investigations.

On physical exam, the patient was capable of achieving a full range of motion in the hip joints with no restrictions or audible clicks. However, active left knee movements were very painful and restricted. Pain was easily elicited with palpation of the knee joint.

**Figure 1:**
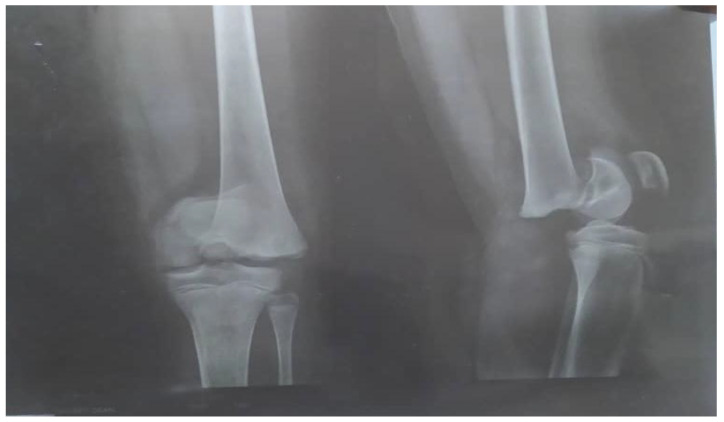
Plain X-ray of the left knee with mechanical non-union fracture.

Bilateral knee arthrocentesis was performed, as well as culture of aspirate, and it all came back negative. A plain radiograph showed a mechanical non-union fracture (Salter–Harris I) of the distal end of left femur bone (Figure 1).

**Figure 2:**
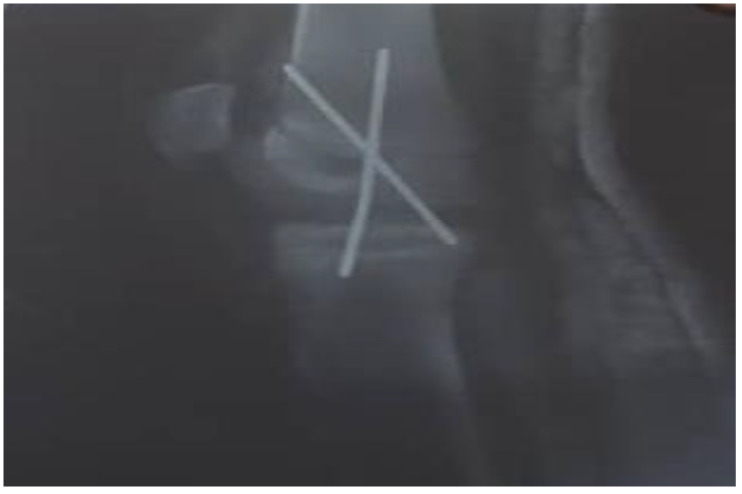
Plain X-ray of the left knee with percutaneous cross-pins for bone stabilization.

The initial therapeutic approach was a percutaneous crossed pins application (Figure 2).

**Figure 3:**
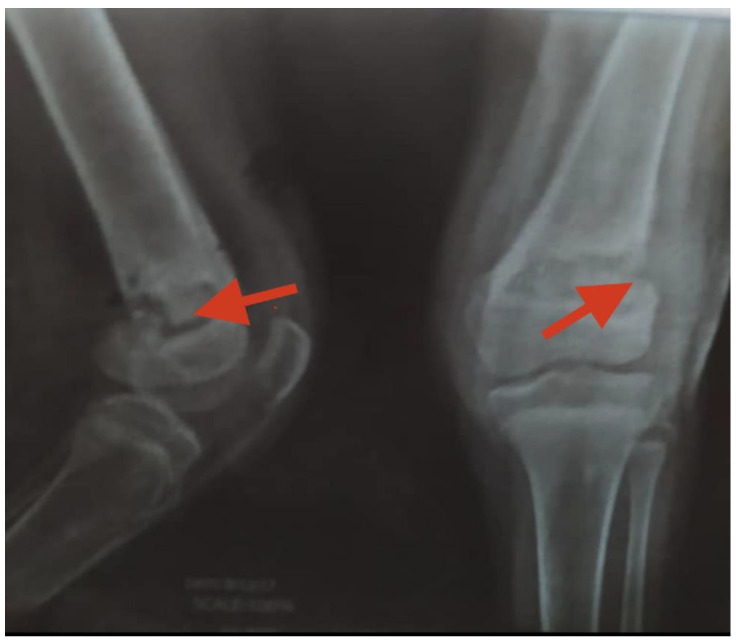
Plain radiograph of the right and left knee with osteomyelitis (red arrows) following the insertion of percutaneous cross-pins.

A prescription of prophylactic antibiotics with IV cefazolin, 1 g every 6 h, and pain medications (NSAIDs) was given, after which the patient was discharged home with cephalexin 500 mg tablets to be taken by mouth (PO) every 12 h for two weeks. Four weeks later, there was no improvement, and the patient returned to the hospital with fever and pain in the left knee not controlled with NSAIDs. A plain radiograph was performed, which revealed osteomyelitis of the distal left femur as shown in Figure 3.

There was also a great reduction of the hyperextended epiphysis; a lateral-based defect with a thin bridging bone anterior-medially was seen on plain X-ray images of the patient’s left knee. A percutaneous bone aspiration of synovial fluid was performed and sent to the lab for testing. The results of the culture of bone aspirate yielded a predominant of staphylococcus microbes.

**Figure 4:**
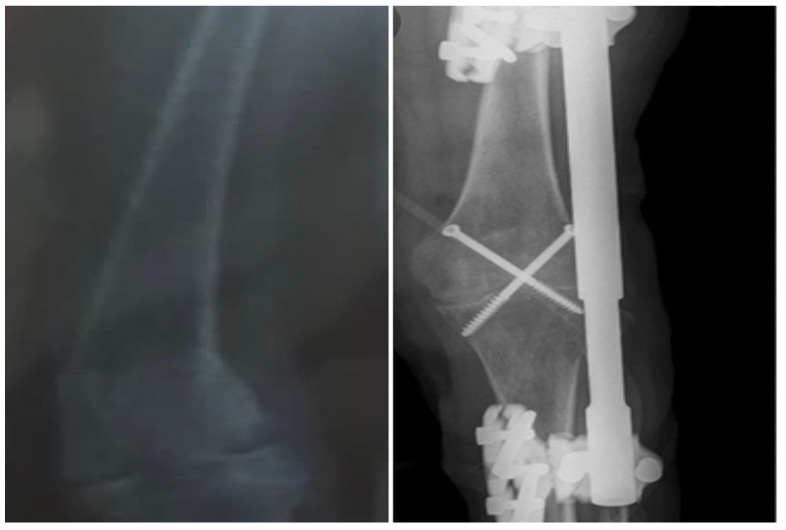
Plain radiograph of the left knee with lateral locking plate after the insertion of new cross-pins following a full recovery from osteomyelitis.

A new treatment plan was made, first involving IV hydration with normal saline, intubation for oxygenation, and pain control, then removal of the previously inserted cross-pins in order to treat the bone infection (osteomyelitis) with 3 g IV of ticarcillin clavulanate every 4 h followed with 1 g IV of vancomycin every 12 h for four weeks. Upon full recovery and completion of the antibiotic regimen, the bone defect was curetted, followed by bone grafting. Later, arthrodesis of the knee joint with double screws and a lateral locking plate was performed to gradually correct the valgus and prevent peroneal palsy (Figure 4).

When the bones were straightened out, a lateral locking plate was applied, followed by more bone grafting, after which the patient commenced a range of motion on the extremities with the help of physiotherapy. The patient could bear weight on both legs with support (crutches) following surgical interventions and antibiotic treatment. He is currently undergoing physiotherapy sessions and is doing well as an outpatient.

## 3. Discussion

The Salter–Harris classification of fractures is based on the involvement of physis, metaphysis, and epiphysis of long bones [12]. These fractures typically occur in the growth plate when children have their growth spurt and when the physis is weak [13].

Salter–Harris fracture is categorized into five groups: the type I (5%) fracture line extends through the physis. It occurs due to external force applied to the knee, splitting the epiphysis from the metaphysis. The type II fracture (most common (75%)), extends through the metaphysis and epiphyseal plate without affecting the epiphysis. The type III (10%) Salter–Harris fracture (Tillaux) is an intra-articular fracture involving the epiphyseal plate and the epiphysis [14]. Type IV (10%) fracture involves the epiphysis, epiphyseal plate, and the metaphysis. Lastly, type V occurs on rare occasions due to a crushing or compression impact on the epiphyseal plate through the epiphysis and physis. The grouping of these fractures and their effects on the physis is based on their severity level. The lower numbers (types I, II, and III) are not severe and have a lower capability of causing growth abnormalities [14]. A study of two pediatric patients [15] with Salter–Harris I fibula fractures in which the fractures were unable to be corrected by closed reduction has been reported: In the first patient, the fracture could not be corrected by closed reduction, but with the reduction of the syndesmosis and application of one syndesmotic screw (which was eventually removed on month 4), the first patient was able to return to sports two months post-op. In the second patient, the fracture was partially corrected with closed reduction, which required opening of the fracture site and the insertion of an intramedullary screw to achieve a complete reduction. The second patient was also able to return to sporting activities 6 months post-op.

The Salter–Harris fracture in our patient (a type I Salter–Harris fracture) was similar to the above-mentioned report; however, in our patient, the fracture line extended through the growth plate due to a strong impact of a longitudinal force on the physis, separating the epiphysis from the metaphysis, as seen in Figure 1. Diagnosis of this type of fracture is based on clinical symptoms/physical examination of the patient with focal tenderness or edema around the growth plate, similar to slipped capital femoris epiphysis.

On several occasions, a fracture diagnosis could be missed on X-rays. Thus, clinicians must always conduct a good physical exam on young patients who present with the symptoms of a fracture to detect these injuries.

When imaging studies fail to detect fractures, muscle sprains and torn tendons are often blamed for most musculoskeletal injuries in children and adolescents [16]. Although Salter–Harris I fractures are commonly treated with closed reduction and casting, special care needs to be employed to avoid damage to the physis. More care is necessary when treating sickle cell patients with fractures, as these groups of patients, to which our patient belonged, are very prone to developing septic arthritis [17].

**Figure 5:**
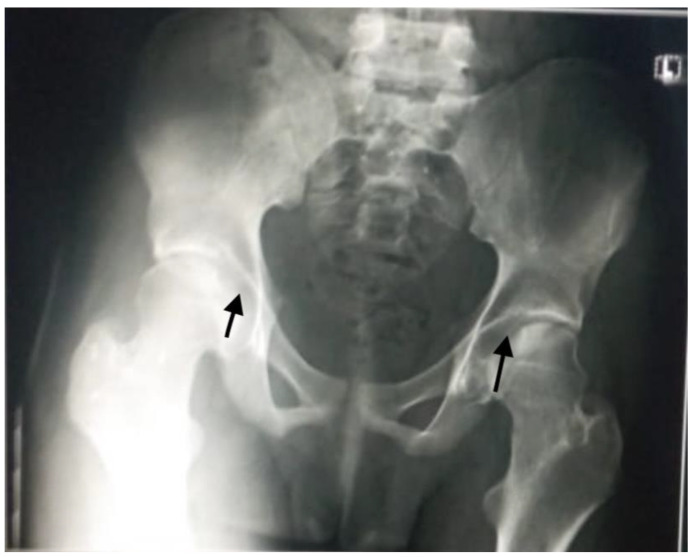
Plain radiograph showing avascular necrosis of the bilateral femoral head (black arrows). Commonly seen in sickle cell disease patients due to poor blood circulation from the disease condition.

Aseptic necrosis of the femoral head is also a common complication of fractures in adolescents with sickle cell anemia as evident in our patient in Figure 5. Another common bone involvement in sickle cell disease patient is osteonecrosis. Osteonecrosis in sickle cell patients tends to affect a larger bone surface area than osteonecrosis due to other etiologies [18]. According to several works of literature, the increased risk of osteonecrosis of the femoral head in our patient group could be linked to an overall reduction in bone mineral density and marrow hyperplasia compared to the general population [18,19].

In general, duration of treatment with antibiotics in sickle cell patients with osteomyelitis often lasts between 4 and 6 weeks [20]. Some clinical trials with extended courses of either parenteral or oral antibiotics have proven a significant improvement in health outcomes with treatment duration of more than 6 weeks [20]. When patients are stable enough for discharge following appropriate treatment of their condition, outpatient IV antibiotics may be given through a peripherally inserted central catheter line.

## 4. Common Bone Involvement in Patients with Sickle Cell Disease

### 4.1. Vaso-Occlusive Crises

Vaso-occlusive crises affect all patients with sickle cell disease, from late infancy and throughout life. The pathogenesis of microvascular occlusion resulting in a painful bone crisis in this patient population is complex, involving activation and adhesion of leucocytes, platelets, and endothelial cells and with hemoglobin S-containing red blood cells [21]. While this process can occur in any organ, it is common in bone marrow, resulting in bone marrow infarction, which is often seen in the medullary cavity or epiphyses [22,23]. The vulnerability of the bone marrow to microvascular occlusion could be explained by marrow hypercellularity leading to impairment of blood flow and regional hypoxia [24]. Common clinical complaints are localized intense body pain accompanied by tenderness, edema, and erythema over the affected part of the body. Fever is also reported. Most patients fully recover from vaso-occlusive crisis with no further complications. However, epiphyses infarction often leads to joint effusions similar to septic arthritis [23,24].

### 4.2. Dactylitis in Small Bones

Dactylitis is common in sickle cell disease children under the age of 7 years. Vaso-occlusion in the bones of the hands and feet results in dactylitis. Common clinical complaints are painful (acute) swelling of one or several digits. Histologically, there is evidence of extensive infarction of the marrow, medullary trabeculae, and the inner layer of the cortical bone, with subperiosteal new bone formation [25]. Most episodes resolve within two weeks when new bone formation becomes evident on bone scans as a ‘moth-eaten’ bony appearance of the involved digits due to cortical thinning and misaligned medullary spaces [22]. Premature fusion with shortened fingers due to epiphyseal infarction is possible but rare [26].

### 4.3. Osteomyelitis in Long Bones

Patients with sickle cell disease are often prone to osteomyelitis caused by *S. aureus*, *Salmonella,* and other gram-negative bacilli. In such cases, positive blood culture, biopsy, or joint aspiration should be used to direct the choice of antibiotics. However, there is a gap in the literature to guide antibiotic selection for sickle cell patients with osteomyelitis according to the 2016 Cochrane review [27]. Therefore, it is always advised that management of sickle cell disease patients with osteomyelitis should be a team approach, which includes an infectious disease specialist to determine the appropriate antimicrobial therapy and duration of treatment based on the individual patient’s needs [28]. A study suggested a first-line treatment of a confirmed or suspected case of osteomyelitis in sickle cell disease patients to be a third-generation cephalosporin to ensure full coverage of gram-positive microbes [29]. Moreover, a combination of vancomycin and ciprofloxacin have also proven to be effective in these patients [29]. However, additional studies are required to further clarify the most effective antibiotic selection for osteomyelitis in sickle cell disease patients’ population.

The rate of sickle cell disease patients’ susceptibility to infections, especially osteomyelitis, has been hypothesized to be linked to hyposplenism, impaired complement activity, and bone necrosis. A recent French study with 299 sickle cell disease patients who were treated and monitored reported a 12% prevalence of osteomyelitis development and a lower prevalence in patients with the Bantu haplotype (commonly seen in Senegalese and Beninese populations) [9,30]. The most common osteomyelitis causative microbial agents are *salmonella*, which we have already discussed.

Management of osteomyelitis in sickle cell disease patients can be very challenging to clinicians as failure to diagnose it may result in a severe and life-threatening infection. A differential diagnosis between painful bone crisis and osteomyelitis is crucial for an early and accurate treatment regimen. Often, osteomyelitis may not be evident until patients fail to recover from a suspected bone crisis following a two-week standard therapy [31]. Blood cultures at the early symptoms of a suspected osteomyelitis and bone crisis in these patients often return sterile. Thus, accurate diagnosis of osteomyelitis in sickle cell patients tends to rely on various imaging studies. In some patients (like in our patient), osteomyelitis presents late, as a more indolent disease process and with abscess formation, in which case it becomes visible on X-ray [32,33].

### 4.4. Avascular Necrosis (Osteonecrosis)

Osteonecrosis occurs due to infarction caused by vaso-occlusion of the long bones’ articular surfaces, particularly the femoral head. In patients with osteonecrosis, the blood supply to the hip joint is interrupted, and the bone begins to die. Osteonecrosis also leads to tiny breaks in the bone and subsequent collapse of the ball of the femoral head. Typical clinical presentations include pain in the hip, thigh, or knee. The hip joints become stiffer, and pain occurs abruptly or after several weeks.

Diagnosis of osteonecrosis may be suspected after a detailed physical examination, history of the illness, and X-rays. Advanced imaging studies are preferred for early diagnosis of osteonecrosis. These may include perfusion MRI, a technique that evaluates blood flow to the bone for non-perfused areas. Another technique is the delayed gadolinium-enhanced MRI of cartilage (dGEMRIC)—a technique used to detect early cartilage breakdown and to map areas of early osteoarthritis or focal cartilage injury [17].

Osteonecrosis treatment should begin with non-surgical management. In some cases, conservative treatment such as bed rest, restriction of physical activities, physiotherapy sessions, and pain medication can resolve the pain and inflammation. When patients fail conservative treatment, surgery for osteonecrosis such as autologous bone-marrow grafting, core decompression, and/or free vascularized fibular grafting may be performed.

### 4.5. Osteoporosis and Osteopenia

Several studies have reported an overall reduction in bone mineral density, attributed to bone marrow hyperplasia, in patients with sickle cell disease [34,35,36,37,38]. Compared to the general population, patients with sickle cell disease have lower bone mineral density values in all bone scan regions [34]. Radiologically, the bone appears as areas of increased translucency of the vertebral bodies, prominence of vertebral trabeculae, and a smooth, biconcave deformity of the vertebrae, also known as ‘fish mouth’ vertebrae as a result of compression by the adjacent intervertebral discs [39]. These patients will later develop vertebral collapse due to osteoporosis or vertebral infarction. Vertebral collapse is often asymptomatic but may cause prolonged or acute pain requiring analgesia and mechanical bone support such as a brace.

It is important to note that impairment of growth in children and adolescents with sickle cell disease is a well-recognized complication of this disease condition [40,41,42,43]. Studies have shown that the main cause of growth impairment in this patient population is due to bone marrow hyperplasia [44]. Bone marrow hyperplasia results in ischemia of the central portion of the vertebral growth plate, causing growth disturbance in the characteristic ‘H’-shaped vertebrae from the compression of the vertebral end plates [36,45]. In addition to bone marrow hyperplasia causing growth impairment in sickle cell patients, a local anoxic event may result in premature closure of epiphyses and asymmetrical growth of the long bons of the extremities [46]. A study [43] conducted a comprehensive assessment of growth, nutrition, and body composition in 36 children with homozygous sickle cell disease. There were no significant differences in height or bone age between the sickle cell disease children considering their age, gender, and race when compared to other healthy children. However, among the children with sickle cell disease, there were significantly delayed skeletal maturation and marked deficits in z-score on bone scan for weight-for-age, height, elbow width, skin fold thickness, and mid-upper arm circumference, indicative of global deficits in energy reserves and in growth [43]. Another study [47,48] showed that sickle cell disease patients have lower levels in body stores for vitamins A, B6, and D compared to the healthy population, which contributes to the disease morbidity in sickle cell disease children.

## 5. Conclusions

In summary, managing fractures in patients with sickle cell anemia requires comprehensive medical care because many complications that affect these patients are primarily musculoskeletal in origin and are the primary source of acute and chronic morbidity. The primary treatment of osteomyelitis in sickle cell patients includes a combination of antibiotics to cover both *Enterobacteriaceae* and *methicillin-resistant staphylococcus* aureus, which proved to be effective in our patient. In some cases, surgical intervention with debridement and bone reconstruction may be necessary. Additionally, management of patients with sickle cell disease requires balanced nutrition, smoking cessation, and close monitoring of any underlying chronic medical conditions associated with the disease.

Although type I and II Salter–Harris fractures often require stabilizing the fractured bones with a cast or splint, type I fractures in sickle cell disease patients are managed the same as types IV and V, which require both medical and surgical interventions. This is because bone fractures in sickle cell disease patients result in osteomyelitis when not adequately managed. Similarly, the management of osteomyelitis in sickle cell disease patients remains problematic, as current imaging techniques often fail to distinguish between osteomyelitis and vaso-occlusive crisis. Such a condition results in more extended hospital stays and the need for more invasive studies, which further increases patients’ risk for sepsis. Hence, the management of fractures in adolescents and children with sickle cell disease remains to be explored.

## Data Availability

Not applicable.
